# The Therapeutic Trip of Melatonin Eye Drops: From the Ocular Surface to the Retina

**DOI:** 10.3390/ph17040441

**Published:** 2024-03-29

**Authors:** Dario Rusciano, Cristina Russo

**Affiliations:** 1 Fidia Research Centre, c/o University of Catania, Via Santa Sofia 89, 95123 Catania, Italy; 2 Department of Biomedical and Biotechnological Sciences, University of Catania, Via Santa Sofia 89, 95123 Catania, Italy; cristina.russo@unict.it

**Keywords:** melatonin, drug delivery, nanotech formulation, glaucoma, dry eye, cataract, diabetic retinopathy, macular degeneration, uveitis, myopia

## Abstract

Melatonin is a ubiquitous molecule found in living organisms, ranging from bacteria to plants and mammals. It possesses various properties, partly due to its robust antioxidant nature and partly owed to its specific interaction with melatonin receptors present in almost all tissues. Melatonin regulates different physiological functions and contributes to the homeostasis of the entire organism. In the human eye, a small amount of melatonin is also present, produced by cells in the anterior segment and the posterior pole, including the retina. In the eye, melatonin may provide antioxidant protection along with regulating physiological functions of ocular tissues, including intraocular pressure (IOP). Therefore, it is conceivable that the exogenous topical administration of sufficiently high amounts of melatonin to the eye could be beneficial in several instances: for the treatment of eye pathologies like glaucoma, due to the IOP-lowering and neuroprotection effects of melatonin; for the prevention of other dysfunctions, such as dry eye and refractive defects (cataract and myopia) mainly due to its antioxidant properties; for diabetic retinopathy due to its metabolic influence and neuroprotective effects; for macular degeneration due to the antioxidant and neuroprotective properties; and for uveitis, mostly owing to anti-inflammatory and immunomodulatory properties. This paper reviews the scientific evidence supporting the use of melatonin in different ocular districts. Moreover, it provides data suggesting that the topical administration of melatonin as eye drops is a real possibility, utilizing nanotechnological formulations that could improve its solubility and permeation through the eye. This way, its distribution and concentration in different ocular tissues may support its pleiotropic therapeutic effects.

## 1. Introduction

Melatonin (N-acetyl-5-methoxytryptamine) is an indoleamine primarily secreted by the human pineal gland during darkness [1] but also produced in various tissues, including the eyes [2]. Melatonin can be considered the logical natural consequence of what could be described as the paradox of life. Life on Earth over the last 2.5–3.0 billion years has evolved in an oxygen-rich atmosphere, developing an aerobic metabolism that, as a byproduct, generates a substantial amount of free oxygen radicals (reactive oxygen species: ROS) [3]. Known as the oxygen paradox [4], it posits that while oxygen is essential for life, it is also toxic to cells and organisms, ultimately causing aging and death.

Most of the oxidative energetic reactions necessary for metabolism and cell survival occur in mitochondria (chloroplasts in plants), which are the primary producers of these highly reactive free oxygen radicals. Approximately 4% of the oxygen used in aerobic metabolism is estimated to be converted to ROS. To survive, cells had to develop an efficient antioxidant system, and melatonin is likely the product of such an evolutionary effort [5]. Indeed, melatonin is already present in unicellular organisms and has been conserved throughout evolution in plants and animals [6].

The primary site of melatonin biosynthesis is in the mitochondria (the major source of ROS generation in living organisms), and its production in other cellular compartments likely derives from this primary site [7,8]. The unique structure of melatonin is responsible for its high efficiency in detoxifying free radicals. Metabolites derived from its antioxidant effect also retain free radical scavenging activity, sometimes even stronger than the original molecule [9]. This potent antioxidant function of melatonin results in the protection of mitochondrial physiology and extends to its other activities, such as anti-inflammatory, neuroprotective, anti-apoptotic, and anti-aging effects [5].

With time, the functions of melatonin have expanded through the development of specific cell receptors. MT1 and MT2 are the main cell membrane receptors, belonging to the class of G protein-coupled receptors and are widely distributed in many tissues [10,11]. MT3 is the low-affinity cytoplasmic receptor for melatonin, identified as quinone reductase 2 [12], belonging to a group of detoxifying enzymes involved in preventing oxidative stress through the inhibition of electron transfer reactions [13].

**Figure 1:**
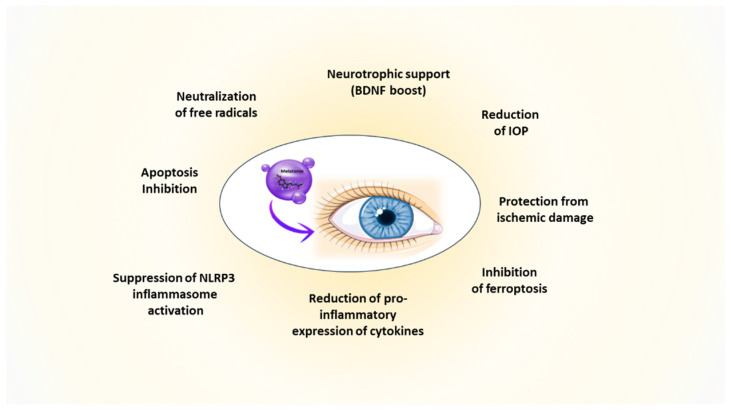
Properties of melatonin and its supposed effects on the eye. Created with Servier Medical Art (https://smart.servier.com) and modified with Microsoft PowerPoint.

Melatonin-producing cells and their related receptors are present throughout the body and in almost all eye tissues, where they play a role in the regulation of eye growth and its dioptric potential, intraocular pressure (IOP), ocular surface physiology, and phototransduction processes [14] (Figure 1). Indeed, the presence of receptor-dependent and -independent functions within the same molecule makes melatonin a very versatile natural drug. This versatility could help rebalance deranged situations leading to ophthalmic pathologies. Here, we aim to explore the possibility of formulating melatonin as eye drops for topical application, to treat or even prevent the development of the most common eye diseases [14], as supported by the research data presented subsequently.

## 2. Topical Formulations of Melatonin

In mammals’ eyes a small amount of melatonin is also present, produced by cells in the anterior segment and the posterior pole, including the retina. The rat retina contains 2 pg/mg, and the rat lens between 0.14 and 0.25 pg/lens. In humans, the aqueous humor contains 6.4 pg/mL of melatonin, which is also present in tears at 200 ng/mL [15]. Therefore, if these naturally present amounts could be increased by eye drops administration, such increased amounts could provide beneficial effects in disease prevention or even treatment of eye pathologies, in which an inflammatory and oxidant component is most often present.

Formulating melatonin as eye drops presents two primary challenges that must be addressed. The first pertains to its stability in aqueous solutions, and the second involves facilitating its transport across the epithelial barrier of the ocular surface.

Melatonin is a small, non-polar molecule with poor solubility in an aqueous solution (0.1 mg/mL or 0.43 mM) and is typically administered orally as a solid, in immediate or modified-release formulations. The use of lipidic encapsulation in nanotechnological formulations allowed higher concentrations of melatonin to be obtained (Table 1). Moreover, such encapsulation can shield the melatonin molecules from spontaneous oxidative events, thus prolonging their stability and efficacy in the formulations. The stability of melatonin in its formulations can be evaluated by HPLC analysis, usually coupled to a mass spectrometry detection, to establish the presence of a native molecule [16,17], or by biological test methods, to establish the presence of an active form of the molecule, using for instance its IOP-lowering effects [18]. Through HPLC analytical methods, it has been demonstrated that melatonin remains stable in aqueous solution for at least 6 months when stored at 4 °C or below, in sterile vacuum-packed vials [19]. Further studies on the stability of aqueous melatonin solutions were conducted at 20 °C and 37 °C at different pH values (1.2–12) for 21 days [20]. No degradation of melatonin was observed during the initial 48 h. However, from day 3 to day 21, a gradual decrease in melatonin occurred at all pH values, though not exceeding 30% of the initial value. Due to its poor stability in aqueous solution, various formulations are being investigated for alternative routes of administration, such as sublingual, transbuccal, ocular, intranasal, or injectable methods [21].

Achieving full solubility of higher concentrations of melatonin would require the use of organic solvents, which are incompatible with ocular application. Therefore, the quest for more tolerable formulations containing higher concentrations of melatonin than achievable in water, and devoid of organic solvents, poses a challenging task in developing a commercial product suitable for topical ocular treatments. Another challenge lies in developing a formulation capable of delivering melatonin beyond the ocular surface. To address this, nanotech ophthalmic systems could be considered to traverse the ocular surface epithelial barrier and reach tissues within the eye globe. Several nanocarriers have been described for ocular drug delivery to different eye segments [22], and some of them have shown promising results in improving ocular bioavailability, reducing the frequency of administration, and minimizing toxic effects on healthy tissues (Table 1).

**Table 1:** Different strategies for topical melatonin formulations and their biological effects.

Formulation	Active Principle	Biological Effect	Effect Duration	Ref
Liposomes + mucoadhesive molecule	MCA-NAT 0.1 mM	IOP reduction 39%	>8 h	[21]
TAT-modified Liposomes	Melatonin 0.1 mM	suppression of pyroptosis and tissue inflammation in vitro in corneal epithelium treated with BCA	nd	[23]
Solid Lipid Nanoparticles (SLN)	Melatonin 2.15 mM	IOP reduction 7 mmHg	24 h	[24]
PLGA polymeric nanoparticles	Melatonin 3.44 mM	IOP reduction 5 mmHg	8 h	[25]
Ethylcellulose nanocapsules	Melatonin 4.3–8.6 mM	Neuroprotection of RGC in an experimental retinal disease model induced in the rabbit’s eye	nd	[26]
PLA nanocapsules	Melatonin 1.29 mM	nd	nd	[27]
Nanomicelles	Melatonin-Agomelatine 17.2 mM–16.4 mM	IOP reduction in ocular hypertensive rats 60%; Neuroprotection of RGC in a model of ocular hypertensive rat	nd	[28]
Nanomicelles	Melatonin 17.2 mM	IOP reduction in normotensive rat 30%	4 h	[29]
Self-Nanoemulsifying Drug Delivery Systems (SNEDDS)	Melatonin 86 mM	biocompatibility only	nd	[30]
Nanofiber-based inserts	Melatonin 0.1, 0.3 and 0.5% *w*/*w*	nd	nd	[31]
Eluting contact lenses	Melatonin ~4 mg/g	increased tear volume; no effects on rabbit IOP	2 h	[32]
Eluting contact lenses	Melatonin 0.16 mM, MCA-NAT 0.17 mM, Agomelatine 0.8 mM	secretagogue effect	4 h	[33]

nd: not detected

### 2.1. Formulations with Liposomes

Liposomes are closed, continuous, vesicular structures composed mainly of phospholipid bilayer(s) in an aqueous environment. In a unilamellar vesicle, the thickness of the lipid bilayer is typically around 5 nm. Therefore, with two sets of bilayers forming a closed sphere and the aqueous space inside the vesicle, the size of a liposome can vary from 20 nm to several micrometers. Alternatively, multilamellar vesicles contain more than one internal aqueous space [23,34]. The main constituents of liposomes are phospholipids, which are amphiphilic molecules. A significant advantage of liposomes is their ability to incorporate hydrophilic molecules into the aqueous compartment, hydrophobic molecules within the lipid membrane, and amphiphilic molecules at the lipid-aqueous interface [35]. This property gives liposomes unique characteristics, making them an ideal transport system in the ophthalmic field.

In a recent study, nanoliposomes (150–200 nm, 6–10 mV zeta potential) were loaded with the melatonin analog 5-methoxycarbonylamino-N-acetyltryptamine (5-MCA-NAT) (100 μM). The formulation also contained a mucoadhesive molecule such as sodium hyaluronate 0.2% (SH) or carboxymethylcellulose 0.5% (CMC) or a thermosensitive amphiphilic poloxamer (PX407 and PX 188; 12/8, *w*/*w*). The aim was to prolong the residence time of the eye drop on the ocular surface and enhance its hypotensive efficacy, as evaluated in normotensive rabbits’ eyes [36]. The results showed that the formulation of 5-MCA-NAT with 0.2% sodium hyaluronate was well-tolerated and the most effective in reducing intraocular pressure (IOP) by 39.13%, compared to all other tested formulations (achieving at most a 29% IOP reduction). The effect lasted more than 8 h. Interestingly, the hypotensive effects of melatonin or 5-MCA-NAT may complement those achieved by the beta-blocker timolol or the alpha-agonist brimonidine. This suggests a mechanism of action different from that exerted by the two classical anti-glaucoma drugs [24]. Since 5-MCA-NAT is a selective MT3 receptor agonist, this suggests the involvement of this receptor in the observed hypotonizing effect [37]. Most recently, melatonin was loaded into liposomes modified with a cell-penetrating peptide, the transactivator of the transcription (TAT) fragment, one of the CPPs derived from the human immunodeficiency virus [25].

### 2.2. Lipid Nanoparticles

Lipid nanoparticles (LNPs) are spherical vesicles with an average size ranging between 40 and 100 nm, composed of a dispersed phase consisting of a solid lipid stabilized with a biocompatible surfactant used as an emulsifier. These lipid nanoparticles are conventionally classified into SLNs (Solid Lipid Nanoparticles) and NLCs (Nanostructured Lipid Carriers) [38]. SLNs are systems comprising a solid lipid matrix, while NLCs are modified SLNs in which a mixture of solid and liquid lipids is present to enhance their drug-loading capacity and stability. Both have been demonstrated as suitable carriers for ocular drug delivery [39].

The ocular hypotensive effect and tolerability of melatonin encapsulated in cationic solid lipid nanoparticles were evaluated in normotensive rabbits. Melatonin-loaded SLNs (0.05% *w*/*v*) were prepared using the QESD method with Softisan100 as the main lipid matrix, and stearic (SA) or palmitic (PA) acid as lipid modifiers, enhancing the physical stability of the nanoparticles. The positive surface charge was achieved by adding a cationic lipid (dodecyl-dimethylammonium bromide) to ensure mucoadhesion and a longer retention time [26]. The effects on intraocular pressure (IOP) after topical administration in albino rabbits’ eyes were compared to an aqueous solution of melatonin over 24 h of observation. The formulation with SA-SLN loaded with melatonin was the most effective in terms of IOP reduction (maximum IOP reduction: 7 mmHg), and its effect lasted approximately 24 h with a significant difference (*p* < 0.01) compared to an aqueous solution of the drug. The ocular tolerability test was evaluated according to a modified Draize test and showed good ocular tolerance of this formulation [26].

### 2.3. Polymeric Nanoparticles

Polymeric nanoparticles (NPs) are colloidal particles (10–1000 nm) composed of biocompatible and biodegradable polymers of synthetic, semisynthetic, or natural origin. The active molecule can be dispersed, encapsulated, or adsorbed on the surface of the nanoparticles. Polylactic-co-glycolic acid (PLGA)—a copolymer of polylactic acid (PLA) and polyglycolic acid (PGA)—is an FDA-approved, highly biocompatible, biodegradable material with mechanical properties modifiable by varying the PLA/PGA ratio and its final molecular weight. It is an excellent drug carrier for ocular administration due to its high hydrophilicity and tolerability. Furthermore, NPs of PLGAs have a high encapsulation efficiency for hydrophilic and hydrophobic drugs, including macromolecules, proteins, peptides, and nucleic acids [40]. PLGA and PLGA-polyethylenglycol (PEG) NPs were used to prepare melatonin-loaded nanocarriers, in which melatonin loading ranged between 44% and 80% [40]. These NPs have been shown to possess adequate characteristics for application to the eye. The hypotensive effect of these NPs was evaluated by measuring IOP during 24 h after instillation in the rabbit eye, as compared to a melatonin aqueous solution at the same concentration (0.08%, *w*/*v*). The maximum IOP-lowering effect (5 mmHg) was obtained using melatonin-loaded PLGA-PEG NPs. The tolerability of these formulations was evaluated following a modified Draize test protocol. NPs did not cause any signs of eye inflammation or tissue changes in the rabbit’s eye [27].

Polymeric nanoparticles can be classified into nanospheres and nanocapsules, both serving as effective drug delivery systems. Nanocapsules function as reservoirs, comprising an oily core where the drug is typically dissolved, surrounded by a polymeric shell that controls the drug’s release profile [28,41,42]. In a rabbit retinal degeneration model, topical formulations of melatonin-loaded ethylcellulose nanocapsules (NCECMEs) demonstrated enhanced transcorneal permeability and neuroprotective effects [29]. These nanocapsules, containing melatonin with an ethylcellulose external cover over an internal oily phase, were prepared using interfacial deposition of preformed polymers. The formulations exhibited favorable properties such as a pH around 5.3, osmolality close to tear fluid, a mean particle size ranging from 150 nm to 180 nm with a narrow size distribution, zeta potential between −25 mV and −30 mV, and encapsulation efficiency around 70%. In vitro release studies showed slower melatonin release from NCECMEs compared to a free melatonin solution. Despite the slower release, NCECMEs facilitated greater penetration without causing irritation during transcorneal application. Stability assessments over 30 days indicated no significant modifications in particle size, polydispersity index, drug content, encapsulation efficiency, zeta potential, and pH. In vivo toxicity and irritation tests after topical application revealed no adverse responses or histopathological changes. Notably, the topical application of melatonin loaded in nanoparticle systems proved more efficient than a melatonin solution, attributed to the prolonged corneal residence time allowing for a more effective and concentrated drug penetration across the cornea after 9 days of treatment. The increased permeation capacity of melatonin observed in NCECMEs correlated with greater neuroprotective and anti-apoptotic effects on retinal ganglion cells, demonstrated by a significant reduction in the apoptosis index and maintenance of retinal integrity compared to the melatonin solution. Indeed, melatonin has been shown to suppress apoptotic pathways in retinal cells, thereby preventing programmed cell death and promoting cell survival. This anti-apoptotic effect contributes to the preservation of retinal function and structure [43]. 

In a different study, Carbone et al. [30] compared hybrid nanocapsules and polymeric nanocarriers for delivering melatonin to the eye (0.03 *w*/*w*). They used FDA-approved polylactic acid (PLA) to prepare nanocapsules without organic solvents. The hybrid nanocapsules, formed by depositing PLA at the interface of a water-in-oil nanoemulsion, included a coating layer with dimethyldioctadecylammonium bromide (DDAB) or cetyltrimethylammonium bromide (CTAB) cationic surfactants. All formulations had sizes < 250 nm and a polydispersity index < 0.2. Cryo-TEM revealed well-defined surfaces in DDAB hybrid nanocapsules. Stability studies showed reversible instability in samples with sediment, dispersible by agitation. CTAB presence led to significant instability. DDAB was chosen for stable nanocapsules with sustained melatonin release (encapsulation efficiency of 87.69%).

### 2.4. Nanomicelles

Surfactants, characterized by hydrophilic ‘heads’ and hydrophobic ‘tails’, spontaneously form micelles in aqueous solutions when concentrations surpass the critical micellar concentration (CMC) [31]. Micelles, aggregates resulting from surfactant self-assembly, play a vital role in drug delivery. The CMC is the minimal concentration needed for amphiphilic molecules to initiate micellization, and it varies for each monomer. Polymer micelle structures, dependent on polymer chemistry, include spherical or cylindrical forms from di-block, tri-block, or graft copolymers [32]. Nanomicelles offer advantages such as ease of preparation, small final size (<200 nm), and high drug encapsulation [32].

In a study by Massimo Dal Monte et al. [33], Soluplus^®^ nanomicelles were used to deliver melatonin and its analog agomelatine, demonstrating a hypotensive effect in both normotensive and hypertensive eyes. Soluplus^®^, a graft copolymer comprising polyvinyl caprolactam-polyvinyl acetate-polyethylene glycol, forms micelles in aqueous solutions above the CMC (7.6 mg/L). The nanomicelles, prepared with a direct dissolution method, exhibited dimensions suitable for ocular instillation, with a size below 200 nm and high homogeneity. The combination of melatonin and agomelatine, used as a systemic antidepressant, showed hypotensive activity. The addition of lipoic acid (0.1% *w*/*v*) as an antioxidant increased the duration of the lowering effect of melatonin and agomelatine by approximately 50%. Moreover, the hypotensive efficacy of topical nanomicellar formulations of melatonin (0.4% *w*/*v*) and agomelatine (0.4% *w*/*v*) in rat eyes was demonstrated using the MCE model [44]. The nanomicellar formulation, prepared with 11.5% Soluplus^®^ in Tris buffer, included lipoic acid (0.1% *w*/*v*) as a stabilizing excipient and antioxidant. Compared to conventional glaucoma medications, melatonin/agomelatine drastically reduced IOP elevation in the MCE model, indicating higher efficacy. Neuroprotective efficacy was evaluated through electroretinography, inflammatory and apoptotic markers, and retinal ganglion cell density. In a different study [45], the ocular hypotensive effect of melatonin incorporated into lecithin/chitosan nanoparticles and Pluronic^®^ F127/chitosan micelles was evaluated. Lecithin/chitosan nanoparticles (241.8 nm, −35.9 mV ± 4.2 mV) were formed through electrostatic interactions, and Pluronic^®^ F127/chitosan micelles (22.7 ± 0.7 mV) were prepared using the direct dissolution method. The addition of chitosan in the micelles shifted the surface charge to strongly positive zeta potential values. Although nanoparticles were not cytotoxic, the permeation effect of F127 was reduced in the presence of chitosan, suggesting that the addition of chitosan in the formulation of Pluronic^®^ F127 micelles was not advantageous.

### 2.5. Self-Nanoemulsifying Drug Delivery Systems

Self-nanoemulsifying drug delivery systems (SNEDDS) were developed and tested for efficient ocular delivery of Sirtuin-1 (SIRT-1) agonists, resveratrol (RSV), and melatonin, for potential applications in diabetic retinopathy treatment. Four types of SNEDDS were made, with different surfactants. Tween^®^ 80-based SNEDDS emerged as the most stable formulation. The optimized formulation, comprising Capryol^®^ PGMC, Tween^®^ 80, and Transcutol^®^ P, loaded with RSV or melatonin, exhibited favorable physico-chemical parameters, including small size, homogeneity, quick emulsion time, transparency, high drug content, mucoadhesion strength, sustained drug release, and cytocompatibility with rabbit corneal epithelial cells. These findings suggest the potential of SNEDDS as effective nanocarriers for ocular drug delivery, addressing challenges associated with stability and bioavailability [46].

### 2.6. Biomechanical Delivery Systems

In recent studies addressing the challenges of enhancing melatonin delivery for ocular applications, three different approaches have been explored. Romeo et al. investigated the use of nanofiber-based inserts for prolonged ocular surface contact time and improved melatonin delivery. They employed electrospinning to prepare poly (vinyl alcohol) (PVA) and poly (lactic acid) (PLA) nanofiber inserts, demonstrating submicron-sized structures with amorphous melatonin, and observed varying release rates based on the polymer type [47]. Serramito et al. synthesized melatonin-eluting contact lenses (CLs) and assessed their impact on tear volume and intraocular pressure. In vitro, both non-functionalized (HEMA) and functionalized (HEMA/APMA) monomers exhibited similar melatonin loading and release. In vivo, these CLs released melatonin over the ocular surface for at least 2 h, increasing tear volume without affecting intraocular pressure [48]. Navarro-Gil et al. explored commercially available hydrogel CLs as a delivery system for melatonin analogs, finding that silicone CLs were more effective in releasing agomelatine and 5-MCA-NAT compared to conventional materials. Preloaded CLs triggered higher tear secretion in rabbits than corresponding eye drops, suggesting their potential as an effective strategy for managing dry eye disease [49]. 

**Figure 2:**
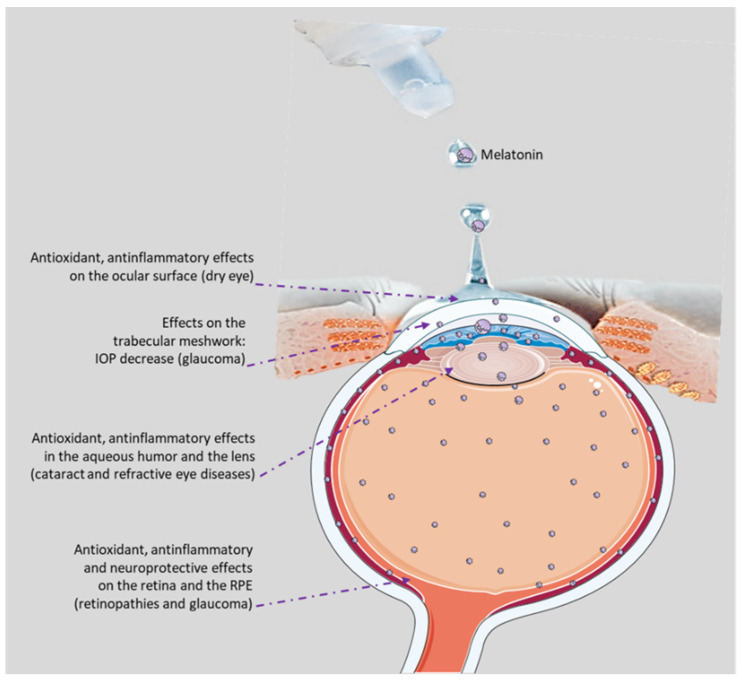
Hypothetical diffusion of melatonin in nanomicelles from the ocular surface to different eye tissues. The figure illustrates a possible ‘vertical’ diffusion, going from the ocular surface through the aqueous humor, the lens, the vitreous body to the retina; and a ‘lateral’ diffusion through the anterior uvea and the local blood circulation [50]. Created with Servier Medical Art (https://smart.servier.com) and modified with Microsoft PowerPoint.

All these studies, taken together, highlight the diverse approaches to optimize melatonin delivery for ocular therapeutic applications (Figure 2).

## 3. Melatonin as a Potential Therapeutic Approach for Dry Eye Disease

Dry eye disease (DED), a prevalent ocular condition affecting approximately 30% of the global population, is characterized by compromised tear film stability, leading to ocular discomfort, visual disturbances, and potential damage to the ocular surface [51]. Recent research has highlighted inflammation as a significant risk factor for DED development [52]. The presence of ROS contributes to inflammation, suggesting that antioxidants could play a crucial role in DED management by mitigating ROS-induced inflammation. In this context, melatonin emerges as a promising therapeutic agent due to its potent antioxidant properties. Melatonin has been demonstrated as an efficient ROS scavenger in SIRC cells and the entire rabbit cornea. It exhibits the capability to inhibit the formation of superoxide anions induced by UV-B exposure or through incubation with fMLP-stimulated autologous macrophages [53]. Further investigations elucidated the molecular mechanisms underlying melatonin’s anti-inflammatory effects. It has been demonstrated that melatonin administration reduced ROS production in HCE cells via the upregulation of heme oxygenase-1 (HO-1), an antioxidant enzyme. This anti-inflammatory effect was further validated in vivo, as melatonin treatment significantly attenuated the mRNA expression levels of NLRP3 and IL-1β in the mouse cornea [54]. Moreover, in a model of corneal neovascularization (CNV) induced in C57BL/6 mice by sodium hydroxide (NaOH), the mice were treated with either vehicle or melatonin eye drops at 0.07 mM. After 7- and 14-days post-burn, the corneas were analyzed. Melatonin-treated mice showed significant inhibition of angiogenesis and reduced corneal epithelial defects compared to controls. Additionally, melatonin reduced the infiltration of inflammatory cells and F4/80+ cells. Quantitative real-time reverse transcription-polymerase chain reaction (qRT-PCR) revealed down-regulation of proangiogenic factors (VEGF, MMP-9), chemokines (MCP-1), and inflammatory molecules (TNF-α, IL-1β, IL-6) in melatonin-treated mice. In vitro experiments using murine peritoneal macrophages confirmed melatonin’s inhibitory effect on these factors. These findings suggest that melatonin can suppress angiogenesis in alkali-injured corneas by inhibiting macrophage infiltration and the secretion of proangiogenic and inflammatory factors [55]. The potential therapeutic benefits of melatonin as an anti-inflammatory agent extended to corneal transplantation. In a mouse model, melatonin administration after corneal allografts effectively inhibited NLRP3 inflammasome activation, thereby reducing the expression of proinflammatory cytokines such as IL-1β, MCP-1, MIP-1, NLRP3, ASC, tumor necrosis factor-alpha (TNF-α), and endothelial growth factor (VEGF)-A. This effect may be mediated by macrophage suppression and T-cell regulation [56]. More generally, melatonin has shown therapeutic utility in reducing graft failure in various types of organ transplantation, including cardiac, bone, otolaryngology, ovarian, testicular, lung, pancreas, kidney, and liver transplantation. The molecular mechanisms behind these effects of melatonin are primarily related to its antioxidant and anti-inflammatory actions, finally reducing oxidative stress and mitigating ischemia-reperfusion injury (IRI). Additionally, melatonin possesses significant anti-inflammatory activity and has been shown to improve the beneficial effects of organ preservation fluids containing indoleamine when enriched with melatonin. This enrichment enhances the immune tolerance of transplanted organs, thereby contributing to the potential reduction of graft rejection following organ transplantation. The multiple pathways behind melatonin action include the upregulation of anti-oxidative enzymes, the neutralization of nitrogen-based toxicants, and the suppression of pro-oxidative enzymes activity. Furthermore, it also discusses the immune-regulatory and immune-suppressive features of melatonin, which are crucial in the context of transplantation medicine. Therefore, the potential of melatonin in reducing graft is due to its antioxidant, anti-inflammatory, and immune-regulatory properties, making it a promising therapeutic agent in organ transplantation [57].

Interestingly, melatonin has been detected in human tears, with concentrations varying throughout the day in accordance with the circadian rhythm [58]. Melatonin has also been identified in New Zealand white rabbit tears, but unlike humans, its production does not follow a circadian pattern [59]. While melatonin itself failed to induce tear secretion in New Zealand rabbits, melatonin analogs such as 5-MCA-NAT, IIK7, and agomelatine have demonstrated significant tear production-enhancing effects [60]. This suggests that the higher affinity of melatonin analogs for melatonin receptors may contribute to their enhanced tear-secretion-inducing capacity [61]. Crooke and collaborators assessed the role of melatonin and its analogs in corneal wound healing. Topical application of melatonin accelerated corneal wound healing in New Zealand rabbits compared to a control condition, mediated by MT2 receptors [59]. Melatonin receptors were also involved in tear secretion mechanisms. It was described that melatonin combined with diadenosine tetraphosphate (Ap4A) synergistically promoted tear secretion when applied topically to the cornea of New Zealand white rabbits. This effect was abolished by luzindole, a melatonin receptor antagonist, supporting the involvement of melatonin receptors in tear secretion [62]. A more recent study aimed to investigate the role and mechanism of melatonin-loaded polymer polyvinyl caprolactam-polyvinyl acetate-polyethylenglycol graft copolymer micelles (Mel-Mic) in dry eye disease [63]. In vitro, Mel-Mic improved the solubility and biological activities of melatonin, reducing apoptosis and ROS in human corneal epithelial cells (HCECs) exposed to hyperosmotic conditions. Mel-Mic also enhanced mitophagy markers and restored impaired mitophagic flux in HCECs. In vivo, Mel-Mic-treated mice that exhibited improved clinical parameters, increased tear production, and reduced goblet cell loss. The protective effects involved PINK1-mediated mitophagy and possibly acted through the MT1 receptor, as demonstrated by antagonist studies. The findings suggest Mel-Mic as a potentially effective treatment for DED. A different model system addressed the efficacy of topical administration of TAT-modified melatonin liposomes (TAT-MT-LIPs) (obtained by chemical grafting of Trans-Activator of Transcription (TAT) onto liposomes) on the ocular surface of rats with Benzalkonium-Chloride (BAC)-induced DED [38]. TAT-MT-LIPs significantly alleviated clinical symptoms by inhibiting tissue inflammation and preventing corneal epithelium and conjunctival goblet cell loss. The study revealed that BAC induces NLRP3/Caspase-1/GSDMD-mediated corneal epithelium pyroptosis, a novel finding. TAT-MT-LIPs efficiently suppressed BAC-induced pyroptosis and inflammation by inhibiting mitochondrial DNA oxidation and subsequent signal transmission. This suggests a potential new target for protecting the corneal epithelium when BAC is used as an eye drop preservative, with TAT-MT-LIPs holding promise as a DED treatment.

In summary, melatonin and its analogs hold promise as therapeutic agents for DED. Their potential benefits include modulating the corneal hydration state, increasing tear secretion, and protecting against ROS-induced inflammation [49]. Further studies employing a multi-level strategy utilizing a combination of melatonin and its analogs acting on distinct receptors are warranted to optimize DED management.

## 4. Melatonin and Cataract

Cataract, a prevalent age-related ocular ailment, involves clouding of the eye lens, a biconvex structure situated between the iris and the vitreous body, responsible for focusing light onto the retina [64]. Comprising water and proteins, the crystalline lens is transparent, enabling unimpeded light transmission to the retina. Structurally, it features a monolayer of cuboidal epithelial cells, a cortex of fibrous cells formed through epithelial cell differentiation, and a nucleus of fibers present from birth [64]. Over the lifespan, fiber cells accumulate high protein concentrations, forming aggregates that scatter light, hindering its focus on the retina [65]. Cataract results primarily from crystallin protein aggregation (α-, β-, and ɣ-crystallins), overexpressed during lens cell differentiation into fiber cells. Despite the necessity of their high protein concentration for maintaining the lens’s refractive gradient and optical performance, aging diminishes the protein biosynthetic capacity of lens fiber cells, limiting damaged crystallin function restoration [66]. Age-related changes in crystallins involve posttranslational structural alterations, including homo- and hetero-oligomeric protein complexes cross-linked through disulfide bridges, a consequence of oxidative stress and glutathione redox imbalance [67]. These changes reduce crystalline protein stability, leading to aggregate formation, uneven protein distribution, light scattering, and subsequent cataract characteristics like reduced transparency, light sensitivity, and visual acuity [67,68]. The role of autophagy and mitophagy in lens organelle degradation is crucial. During maturation, differentiated fiber cells lose internal organelles, including the mitochondria, nuclei, Golgi apparatus, and endoplasmic reticulum [69,70]. Mitochondrial degradation triggers the maturation phase, and maintaining mitochondrial homeostasis in lens epithelium is vital for cell viability. Incomplete removal of damaged mitochondria leads to undesirable light scattering in cortical fibers [71].

Melatonin plays a pivotal role in inhibiting cataract formation through its diverse properties. Extensive research has highlighted its presence in crucial ocular components such as the retina, iris, ciliary body, crystalline lens, and lacrimal gland [72,73,74,75,76]. A notable study published in 1994 provided early insights into melatonin’s antioxidative role, demonstrating its capacity to inhibit cataract formation in newborn rats with glutathione deprivation-induced cataract. The findings indicated a significant reduction in cataract incidence, emphasizing melatonin’s protective effects against oxidative stress, potentially attributed to its role in scavenging free radicals and stimulating glutathione production [77]. Building on these observations, subsequent research explored melatonin’s impact on selenite-induced cataracts in rat eyes. The study revealed a significant decrease in cataract incidence following melatonin treatment, accompanied by favorable alterations in oxidative stress markers and enhanced antioxidant enzyme activities [78]. Further elucidating the protective mechanisms of melatonin, Bai et al. in 2013 investigated its role in human lens epithelial cells. The study demonstrated melatonin’s superior efficacy in preventing H_2_O_2_-induced damage compared to vitamin E. Melatonin exhibited its protective effects through the activation of antioxidant enzymes and the PI3K/Akt signaling pathway, ensuring cellular survival [79]. More insights into melatonin’s involvement in ocular lens organelle degradation were provided by addressing the role of autophagy. The presence of autophagic vesicles containing mitochondria in various lens compartments was demonstrated, underscoring the importance of mitophagy in maintaining lens homeostasis and preventing cataract formation [80]. Jenwitheesuk et al. in 2014 expanded the understanding of melatonin’s multifaceted role, linking it to aging, neurodegeneration, energy metabolism, epigenetics, autophagy, and circadian rhythm pathways. Their study suggested that melatonin’s impact on autophagy depended on cellular conditions, either inducing or inhibiting it, accordingly [81]. In a more recent investigation, melatonin has been found to inhibit ferroptosis and delay age-related cataract by regulating specific pathways. This inhibition of ferroptosis by melatonin is achieved through the activation of SIRT6/p-Nrf2/GPX4 and SIRT6/NCOA4/FTH1 pathways. It was observed that melatonin rescued the survival of cells exposed to UVB-induced ferroptotic stress, and this effect was attributed to the inhibition of ferroptosis. Melatonin’s mechanism of action involved neutralizing lipid peroxidation toxicity, thereby protecting cells against ferroptotic stress in vitro and delaying cataract formation caused by UVB exposure in rats. These findings suggest that melatonin plays a significant role in modulating ferroptosis, highlighting its potential therapeutic implications for conditions associated with ferroptosis, such as age-related cataracts [82]. Examining melatonin’s ability to counteract oxidative damage, Lledo et al. [83] shed light on its regulatory role in Nrf2 and NLRP3 inflammasome activity. Melatonin’s interventions included preventing ROS generation, promoting antioxidant capacity, and attenuating inflammatory and cytotoxic effects induced by oxidative stressors. These findings underscored melatonin’s potential as a therapeutic agent for cataract prevention [83]. In conclusion, the collective body of research portrays melatonin as a promising candidate for addressing age-related cataracts and stress-related eye diseases. Its diverse properties, encompassing antioxidative, mitochondrial-protective, autophagy-modulating, anti-inflammatory, and anti-angiogenic functions, underscore its potential as a valuable component in the treatment and prevention of cataracts [78,84].

## 5. Melatonin, a Versatile Molecule in the Retina

Melatonin, a hormone primarily synthesized by the pineal gland and additionally produced in the retina, originates from the amino acid tryptophan found in the bloodstream [85]. Its production and release follow a daily rhythm, peaking at night and dipping during the day [86]. Enzymes in the liver, kidneys, and central nervous system then break down circulating melatonin [86]. Vertebrate retinas express melatonin receptors known as MT1 and MT2 [87]. These receptors, when activated by melatonin, function as neurohormones or neuromodulators in the retinal pigment epithelium (RPE). MT1 and MT2 inhibit the adenylate cyclase pathway through the Gi protein (G protein inhibitor) [88]. These receptors can exist as pairs (homodimers) or mixed pairs (heterodimers). In photoreceptor cells, melatonin’s effect is primarily mediated by heterodimeric MT1/MT2 receptors, which activate a different signaling pathway involving other molecules like PLC and PKC [89]. The MT1 melatonin receptor couples with pertussis toxin-sensitive Gi and -insensitive Gq/11 G proteins, inhibiting forskolin-stimulated cAMP, protein kinase A signaling, and CREB phosphorylation. Additionally, the MT1 receptor enhances phosphorylation of mitogen-activated protein kinase 1/2 and extracellular signal-regulated kinase 1/2, while also increasing potassium conductance through Kir inwardly rectifying channels. Activation of the MT2 melatonin receptor inhibits forskolin-stimulated cAMP production and cGMP formation, activates protein kinase C (PKC) in the suprachiasmatic nucleus (SCN), and reduces calcium-dependent dopamine release in the retina [90]. The initial insights into melatonin’s role in countering retinal pathologies emerged from a study on frog photoreceptors. Exposing these photoreceptors to a brief light source resulted in the generation of ROS, a phenomenon effectively neutralized by low doses of melatonin [91].

### Melatonin’s Effects on Retinal Function

Studies in mice suggest that melatonin, through a pathway involving PKC, can boost the amplitude of the a- and b-waves in the scotopic electroretinogram (ERG), a measure of retinal function in low light [92]. Conversely, in a double-blind placebo-controlled study involving 13 healthy volunteers who ingested 10 mg of pineal melatonin at 4 p.m., the b-wave amplitude exhibited a significant reduction, whether measured in dark or light conditions. This suggests that melatonin may have complex and context-dependent effects on retinal signaling and possesses the ability to transmit signals not only in the pineal gland but also in the retina [93]. Another study explored the influence of melatonin timing on chick ERG. It was found that the circadian rhythm of the chick electroretinogram (ERG) is under the influence of the indoleamine hormone melatonin [94]. To explore whether the melatonin concentration or the timing of administration induces distinct effects on ERG parameters, experiments were conducted with melatonin administered at different times of the day. Circadian rhythms of a- and b-wave implicit times and amplitudes were evident under both light:dark (LD) and continuous darkness (DD) conditions. Intramuscular administration of melatonin at doses of 1 mg/kg and 100 ng/kg resulted in decreased a- and b-wave amplitudes and increased a- and b-wave implicit times. This effect was more pronounced than that observed with 1 ng/kg melatonin, which had minimal impact compared to saline controls. Furthermore, the influence of 1 mg/kg and 100 ng/kg melatonin on a- and b-wave amplitudes in LD, and on b-wave amplitude in DD, was more prominent during the night (ZT/CT 17) compared to the day (ZT/CT 5). The fold change in b-wave implicit time over controls was higher during the day (ZT/CT 5) than at night (ZT/CT 17). These findings suggest that melatonin may contribute to regulating a diurnal and nocturnal functional transition in the retina, potentially through the modulation of a retinal clock [94]. Overall, these studies raise intriguing questions about how melatonin interacts with the retina. While promoting PKC activity in mice might enhance low-light vision, other studies show reduced activity in humans and dose-dependent effects in chicks. Further research is needed to understand the nuanced role of melatonin in retinal function and the potential for individual differences.

## 6. Melatonin and Age-Related Macular Degeneration (AMD)

Melatonin exhibits significant implications for AMD, a condition intricately linked to oxidative stress in the retina, stemming from elevated oxygen consumption rates and exposure to natural or artificial light sources [95]. AMD, characterized by clinical hallmarks like drusen and extracellular depositions indicative of inefficient retinal metabolism, is strongly associated with oxidative stress [96]. Notably, AMD patients present lower circulating melatonin levels compared to their healthy counterparts [97]. Melatonin, renowned for its role as a potent ROS scavenger, emerges as a crucial player in retinal pathophysiology, effectively counteracting oxidative damage to retinal cells. A study administering melatonin to AMD patients demonstrated promising outcomes, with 55 out of 100 patients experiencing stabilized visual acuity at the six-month follow-up [98].

In an in vitro model of RPE, melatonin showcased its antioxidative protective effects by directly or indirectly activating its receptors [99]. The study revealed a concentration-dependent modulation of this protective effect, with luzindole, a melatonin receptor antagonist, blocking the effect at lower concentrations (up to 10^−8^ M) but proving ineffective at higher concentrations (above 10^−6^ M) [99]. Cellular aging, often associated with telomere shortening triggered by oxidative environmental factors, is a contributing factor in AMD due to RPE senescence [100]. Melatonin, an antioxidant molecule, has the potential to indirectly modulate telomere shortening [101]. In the retina, melatonin has been shown to down-regulate hTERT expression (telomerase catalytic subunit) and stimulate telomerase activity, thereby preventing telomere shortening [102].

The breakdown of the blood-retinal barrier (BRB) is a hallmark of AMD, and melatonin has been found to counteract structural and functional changes in the BRB associated with AMD development [103]. In a murine model mimicking Nonexudative AMD (NE-AMD), melatonin prevented oxidative stress-induced alterations in Bruch’s membrane structure and mitigated the decrease in RPE melanin and melanosome content, essential for light absorption. Moreover, melatonin significantly impeded the increase in temporal RPE mitochondria superoxide content induced by the model [104].

Mitochondrial impairment in human RPE cells affected by AMD is linked to oxidative/nitrosative stress, contributing to AMD development. Melatonin’s effect on mitochondrial function plays a crucial role in reducing oxidative stress, inflammation, and apoptosis in the retina. These findings underscore melatonin’s potential as a preventive and therapeutic agent for AMD [105].

Furthermore, recent research suggests that melatonin plays a role in regulating immune homeostasis in the retina through regulatory T cells (Tregs) [106]. Studies have shown that melatonin can restore retinal integrity and ameliorate retinal degeneration in mouse models of retinopathy induced by NaIO3. This effect may be attributed to the conversion of pro-inflammatory M1 macrophages to anti-inflammatory M2 macrophages, promoting tissue repair and immune regulation. Melatonin treatment also upregulates TET2 gene expression and demethylates NT5E, potentially enhancing Treg recruitment in the retinal microenvironment. These findings suggest that melatonin could modulate immune responses and provide a promising therapeutic strategy for AMD.

However, while these studies paint a promising picture, it is crucial to remember that research on melatonin’s role in AMD is still ongoing. More extensive clinical trials are needed to fully understand its efficacy and safety as a potential therapeutic intervention. In conclusion, melatonin’s multifaceted approach to combating oxidative stress, cellular aging, and mitochondrial dysfunction positions it as a potential contender in the fight against AMD. While further research is required, its preliminary promise encourages exploration of its potential to protect our vision from this debilitating disease.

## 7. Melatonin Effects on Metabolic Syndrome and Diabetic Retinopathy

Melatonin has pleiotropic effects on metabolic dysfunctions and gut microbiota. Aging-induced changes in gut microbiota composition and bile acid patterns are known to contribute to hepatic lipid dysmetabolism [107]. However, oral melatonin treatment may reverse these alterations by inhibiting the gut microbiota-mediated deconjugation of bile acids, thereby reducing hepatic lipid dysmetabolism [107]. Additionally, melatonin supplementation has been shown to improve lipid dysmetabolism in the ileum and white adipose tissue by modulating gut microbiota and angiopoietin-like 4 expression, offering potential therapeutic benefits for metabolic syndrome [108]. Furthermore, melatonin’s regulation of circadian rhythms and metabolic processes is evident in studies demonstrating its ability to restore the diurnal rhythmicity of clock genes, lipid profiles, and gut microbiota composition in mice fed a high-fat diet [109]. This suggests that melatonin may play a crucial role in regulating metabolic homeostasis, particularly in the context of obesity. Moreover, dietary melatonin supplementation has been shown to mitigate the adverse effects of a high-fat/high-sugar diet on body weight, lipid metabolism, and organ function in mice, indicating its potential as a therapeutic agent for managing metabolic syndrome [110]. Additionally, melatonin prevents lipid accumulation and gut microbiota dysbiosis induced by a high-fat diet in mice, suggesting its protective effects against obesity-related metabolic disorders [111]. These findings underscore the importance of melatonin in modulating gut microbiota composition and metabolic processes to prevent or mitigate the development of metabolic syndrome. Furthermore, dysregulation of circadian rhythms has been implicated in diabetes mellitus, highlighting the potential role of melatonin in regulating carbohydrate metabolism and glycemic control [112]. Therefore, melatonin supplementation or analogues may hold promise in diabetes management and prevention by restoring circadian rhythmicity and regulating carbohydrate homeostasis [112]. Overall, melatonin emerges as a multifaceted regulator of metabolic processes and gut microbiota composition, offering potential therapeutic avenues for managing metabolic syndromes and related conditions. 

Among the metabolic dysfunctions, diabetes plays a prominent role, and diabetic retinopathy is a feared consequence of poorly controlled diabetes. Diabetic retinas are associated with abnormal vascular changes such as dilatation and deformation. HIF-1a, VEGF-A, and PEDF were all increased because of diabetic injury. Melatonin decreased retinal nitrotyrosine and malondialdehyde levels, showing antioxidative support. The vasculo-modulator cytokines are decreased accordingly by melatonin therapy. Melatonin normalized the retinal vascular changes as well [113]. In a model of early T2DM in adult rats, animals were subcutaneously implanted with a pellet of melatonin. At 12 weeks of treatment, melatonin, which did not affect glucose metabolism in control or diabetic rats, prevented the decrease in the electroretinogram a-wave, b-wave, and oscillatory potential amplitude, and the increase in retinal lipid peroxidation, NOS activity, TNF-α, Muller cells glial fibrillary acidic protein, and vascular endothelial growth factor levels. In addition, melatonin prevented the decrease in retinal catalase activity. These results indicate that melatonin protected the retina from the alterations observed in an experimental model of DR associated with type 2 diabetes [114]. In diabetic Sprague Dawley rats, melatonin (10 mg/kg daily, i.p.) was administered from the induction of diabetes and continued for up to 12 weeks and retinal samples were collected. The retina of diabetic rats showed depletion of glutathione and downregulation of glutamate cysteine ligase (GCL). Melatonin significantly upregulated GCL by retaining the nuclear factor erythroid 2-related factor 2 (Nrf2) in the nucleus and stimulating Akt phosphorylation. The production of proinflammatory cytokines and proteins, including interleukin-1 β (IL-1β), TNF-α, and inducible nitric oxide (NO) synthase (iNOS), was inhibited by melatonin through the NF-κB pathway. At 12 weeks, melatonin prevented the significant decrease in the ERG a- and b-wave amplitudes under the diabetic condition. These results suggest potent protective functions of melatonin in diabetic retinopathy [115]. In diabetic rats, melatonin (20 mg/kg) was given orally for 7 weeks in diabetic rats starting 1 week after the induction of diabetes. Diabetes significantly increased the mean scores of fluorescein leakage, and MDA and ROS levels compared to the control group. Treatment of the diabetic rats with melatonin for 7 weeks prevented the alterations induced by diabetes in comparison with the diabetic control group. Based on these findings, it can be concluded that melatonin might have beneficial effects in prevention of diabetic retinopathy [116]. Under starvation, mitochondria can fuse with each other to maintain bioenergetic efficiency. When there is a nutrient overload, fragmenting mitochondria is a way to store nutrients to avoid energy waste. In cultured 661W cells, a photoreceptor-derived cell line, hyperglycemic conditions triggered an increase in the expression of dynamin-related protein 1 (DRP1), a protein marker of mitochondrial fission, and a decrease in mitofusin 2 (MFN2), a protein for mitochondrial fusion. Further, these hyperglycemic cells also had decreased mitochondrial Ca^2+^ but increased cytosolic Ca^2+^ Treating these hyperglycemic cells with melatonin averted hyperglycemia-altered mitochondrial fission-and-fusion dynamics and mitochondrial Ca^2+^ levels. We gave melatonin to streptozotocin (STZ) -induced type 1 diabetic mice by daily oral gavage and determined the effects of melatonin on diabetic eyes. We found that melatonin was not able to reverse the STZ-induced systemic hyperglycemic condition, but it prevented STZ-induced damage to the neural retina and retinal microvasculature. The beneficial effects of melatonin in the neural retina in part were through alleviating STZ-caused changes in mitochondrial dynamics and Ca^2+^ buffering [117]. The following study demonstrated decreased serum melatonin in pre-diabetic rats, as well as increased concentration of retinal lipid peroxidation TBARS (thiobarbituric acid reactive substances), and protein oxidation (advanced oxidation protein products, AOPP). Oral supplementation with melatonin (85 μg/animal/day) caused melatonin and HDL cholesterol levels to rise in treated rats and reduced levels of fasting serum glucose and fructosamine. Finally, supplementation with melatonin reduced concentrations of TBARS, AOPP, iNOS, VEGF, and MMP9 to a significant level. Thereby exerting an overall positive effect on oxidative stress and pro-angiogenic signaling in the pre-diabetic retina. Thus, oral melatonin might be considered in an early treatment or in the prevention of retinal changes associated with pre-diabetes [118]. Reactive gliosis and pro-inflammatory cytokine production by Müller cells contribute to the progression of DR. In this study, melatonin inhibited the gliosis activation and inflammatory cytokine production of Müller cells in both in vitro and in vivo models of DR. Furthermore, melatonin inhibited Müller cell activation and pro-inflammatory cytokine production by upregulating the long noncoding RNA maternally expressed gene 3/miR-204/sirtuin 1 axis [119]. In the following study, melatonin inhibited oxidative stress and inflammation by enhancing the expression and activity of silent information regulator factor 2-related enzyme 1 (Sirt1) both in in vitro and in vivo models of DR, and the Sirt1 inhibitor EX-527 counteracted melatonin-mediated antioxidant and anti-inflammatory effects on Müller cells. Moreover, melatonin-enhanced Sirt1 activity through the maternally expressed gene 3 (MEG3)/miR-204 axis, leading to the deacetylation of the Sirt1 target genes fork-head box o1 (Foxo1) and nuclear factor kappa B (NF-κB) subunit p65, eventually contribute to the alleviation of oxidative stress and inflammation. The study revealed that melatonin promotes the Sirt1 pathway, thereby protecting the retina from DM-induced damage [120]. In this other study, we characterized the protective effects of melatonin on the inner blood–retinal barrier (iBRB), as well as the possible mechanisms in experimental DR. Results showed that in diabetic rat retinas, the leakage of iBRB and the expression of inflammatory factors (VEGF, TNF-α, IL-1β, ICAM-1, and MMP9) increased dramatically, while the expression of tight junction proteins (ZO-1, occludin, JAM-A, and claudin-5) decreased significantly. The above changes were largely ameliorated by melatonin. Melatonin could maintain the iBRB integrity by upregulating the expression of tight junction proteins [121]. To evaluate the effects of melatonin on DR, the role of melatonin in retinal angiogenesis and the inner blood-retina barrier (iBRB) was investigated under high glucose conditions in vitro and in vivo. Melatonin administration ameliorated high glucose-induced iBRB disruption, cell proliferation, cell migration, invasion, and tube formation, and decreased the expression levels of VEGF, MMP-2, and MMP-9. Furthermore, melatonin treatment increased the level of autophagy but decreased the expression levels of inflammation-related factors under high glucose conditions. It was found that melatonin inhibited the activation of the Wnt/β-catenin pathway following DR. Melatonin exerts protective effects on experimental DR via inhibiting the Wnt/β-catenin pathway by, at least partially, alleviating autophagic dysfunction and inflammatory activation [122].

## 8. Melatonin and Retinal Ischemia

Melatonin, the sleep hormone, shows promise in protecting the retina from damage caused by insufficient oxygen and blood flow. In fact, melatonin’s antioxidant, anti-inflammatory, anti-angiogenic, neuroprotective, and vasculo-protective properties work together to provide a comprehensive approach to protecting the retina from damage caused by ischemia [123]. Several experimental model systems corroborate the efficacy of melatonin in mitigating the pathological consequences of retinal ischemia.

In an experiment with C57BL/6 mice, a sudden increase in pressure inside the eye was induced to create a temporary lack of blood flow in the retina. Prior to, during, and after this induced ischemia, mice were injected with either melatonin or a neutral substance. Twenty-four hours after the ischemia, the levels of HIF-1α and GFAP, important proteins related to tissue response, peaked in the affected retina. When the ischemic retina was treated with melatonin, the overexpression of HIF-1α and GFAP was inhibited. Additionally, two weeks after the ischemia, a greater number of retinal ganglion cells (RGCs) survived in the retinas treated with melatonin compared to those treated with the neutral substance. Melatonin treatment led to increased survival of RGCs in the ischemic mouse retina. This protective effect of melatonin appears to be achieved by preventing the stabilization of HIF-1α and reducing the activity of glial cells in the ischemic retina [124].

Another study aimed to explore the effects of melatonin on retina neovascularization (RNV) and neuroglia in a mouse model of oxygen-induced retinopathy (OIR), a condition mimicking retinal hypoxia in premature infants. The findings indicated a decrease in leakage from retinal blood vessels in OIR mice following melatonin treatment. In response to oxygen-induced injury, there was a reduction in the density of astrocytes along with changes in their structure and function. The activation of the HIF-1α-VEGF pathway observed in the retinas of OIR mice was suppressed in those treated with melatonin. Melatonin not only prevented abnormal blood vessel growth but also protected neuroglial cells and exhibited anti-inflammatory effects by inhibiting the HIF-1α-VEGF pathway in OIR retinas. This suggests that melatonin could be a promising treatment for retinopathy of prematurity (ROP) [125,126].

In another particular investigation, researchers utilized a mice model of oxygen-induced retinopathy (OIR) to simulate conditions akin to retinal hypoxia and ischemia. Their aim was to delve deeper into the protective impact of melatonin on neonatal retinal nerve cells. Melatonin played a role in maintaining the typical structure and thickness of the inner retina, and it safeguarded populations of inner retinal neurons in areas deprived of blood flow by preventing their programmed cell death. Furthermore, melatonin ameliorated visual impairment. Following melatonin treatment, the heightened levels of cleaved caspase-3 and Bax proteins, indicative of cellular apoptosis in response to hypoxia and ischemia, decreased, while the reduced levels of Bcl-2 protein increased. Moreover, melatonin promotes the expression of neurotrophic factors in the retina, fostering the growth, survival, and function of retinal neurons. This neurotrophic support enhances the resilience of retinal cells against various insults [127]. More specifically, melatonin boosted the levels of BDNF (brain-derived neurotrophic factor) and activated downstream pathways involving phospho-TrkB/Akt/ERK/CREB. These findings suggest that melatonin’s anti-apoptotic and neuroprotective effects on inner retinal neurons following hypoxia and ischemia are, at least partially, attributed to its modulation of the BDNF-TrkB pathway [127].

In tandem with these experimental findings, animal model studies accentuated the significance of melatonin in counteracting retinal hypoxia. By attenuating vascular VEGF and NO levels (both of which were elevated in hypoxic animals), melatonin demonstrated its efficacy in reducing retinal permeability and mitigating associated edema. These findings bolster the premise that melatonin supplementation could hold therapeutic promise in managing retinal complications stemming from hypoxia [128,129].

Collectively, these insights reinforce the notion that melatonin serves as a formidable ally in shielding the retina against ischemic insults, offering a multifaceted strategy encompassing neuroprotection, anti-inflammatory action, and vascular stabilization. By targeting various facets of retinal pathology, melatonin emerges as a promising therapeutic intervention for mitigating the detrimental effects of retinal ischemia. Indeed, an insightful clinical study (NCT04005222) involving 28 patients with ocular ischemic syndrome (OIS) revealed compelling results. The study demonstrated that a one-month oral supplementation with selenium (0.1 mg/kg twice daily) and melatonin (0.5 mg/kg twice daily) led to a notable reduction in malondialdehyde (MDA) levels and a significant increase in glutathione (GSH) levels in the aqueous humor of the treated patients. These findings underscore the potential of selenium and melatonin supplementation in mitigating oxidative stress and enhancing antioxidant defenses in individuals with OIS [130]. 

## 9. Melatonin and Glaucoma

Glaucoma, the second leading cause of visual impairment and blindness globally, manifests as a multifactorial disease marked by the progressive degeneration of retinal ganglion cells (RGCs) and subsequent optic nerve damage, culminating in blindness [131,132]. Central to its pathology is the elevation of intraocular pressure (IOP), predominantly caused by increased resistance to aqueous drainage through the trabecular meshwork (primary open-angle glaucoma) and obstruction of the drainage pathway by the iris (primary closed-angle glaucoma) [133,134]. Notably, high IOP correlates with RGC death, underscoring the significance of IOP management in glaucoma treatment [135,136]. Consequently, current therapeutic strategies predominantly revolve around IOP control through pharmacological and surgical interventions [136,137,138]. However, the side effects associated with glaucoma medications prompt continual exploration for alternative therapies.

Melatonin emerges as a promising avenue for glaucoma management due to its involvement in IOP regulation [12,139]. Dysregulation of ocular melatonin levels and signaling correlates with elevated nocturnal IOP and RGC loss [140]. Melatonin’s actions primarily occur through specific receptors (MT1, MT2, and putative MT3), abundantly expressed in retinal cells and the ciliary epithelium [87,141]. Stimulation of these receptors, particularly in the ciliary body, leads to reduced aqueous humor secretion, thereby lowering IOP [142].

In animal models of glaucoma, such as DBA/2J mice, decreased expression of melatonin receptors, particularly MT2, is associated with disease progression, while aqueous humor melatonin levels remain unchanged [143]. Furthermore, suppression of the MT1 receptor correlates with photoreceptor and RGC loss in mice [144,145]. Human studies also corroborate the role of melatonin in glaucoma, with high IOP patients exhibiting elevated aqueous humor melatonin levels compared to normotensive individuals [139,146]. Moreover, urinary levels of melatonin metabolites correlate with glaucoma severity, indicating its potential as a biomarker [147,148]. Notably, disruptions in circadian rhythms, including sleep patterns, are associated with RGC loss in glaucoma patients [149].

Experimental evidence suggests that exogenous melatonin and its analogs effectively lower IOP in animal models and human patients with glaucoma [37,150,151]. These effects are mediated, in part, by modulating adrenergic receptors involved in aqueous humor production [152]. Notably, nanomicellar formulations of melatonin and its analogs demonstrate prolonged hypotensive effects and neuroprotection in glaucoma models [33,44]. Additionally, combined melatonin formulations exhibit enhanced neuroprotective effects against glutamate-induced cytotoxicity, oxidative stress, and nitrosative stress, all implicated in glaucoma pathology [153].

Human studies further support the therapeutic potential of melatonin in glaucoma, with oral melatonin administration reducing IOP and enhancing surgical outcomes during cataract surgery [154]. Agomelatine, an analog of melatonin, also demonstrates IOP-lowering effects and neuroprotection in humans [155] and animal models [156]. Moreover, melatonin’s antioxidant properties protect against retinal damage induced by glutamate excitotoxicity and oxidative stress, both implicated in glaucoma progression [157,158,159].

Melatonin has demonstrated effectiveness also in treating pseudoexfoliative pigmentary glaucoma. Its strong ability to suppress melanin production, its antioxidant properties, and its capacity to lower intraocular pressure make it a valuable treatment for this condition. In cases of pseudoexfoliative glaucoma, where environmental factors contribute to the development of pseudoexfoliative material, melatonin’s antioxidant and pressure-lowering characteristics may provide additional therapeutic advantages [160]. 

Finally, melatonin has also shown neuroprotective effects in normotensive glaucoma (NTG), an effect likely achieved through the modulation of the NRF2/p53/Sirt1 redox-sensitive signaling pathway [161].

Given the neurodegenerative nature of glaucoma, therapeutics with neuroprotective properties, like melatonin, hold significant promise [162,163]. Melatonin’s multifaceted actions, ranging from IOP regulation to antioxidant and neuroprotective effects, position it as a valuable candidate for glaucoma management, warranting further exploration in clinical settings.

## 10. Melatonin and Uveitis

Uveitis, a sight-threatening ocular inflammatory condition, presents significant challenges in its management. Uveitis encompasses a diverse group of intraocular inflammatory disorders characterized by inflammation of the uvea, retina, and vitreous. The pathogenesis of uveitis involves a complex interplay of immune-mediated processes, cytokine dysregulation, and oxidative stress, resulting in inflammation, tissue damage and vision impairment [164]. In recent years, melatonin, also endowed with potent anti-inflammatory and immunomodulatory properties [165], has emerged as a potential therapeutic candidate for uveitis. Melatonin exerts pleiotropic effects through its interactions with melatonin receptors MT1 and MT2, and its modulation of NF-κB signaling. These actions contribute to the downregulation of pro-inflammatory cytokines, such as TNF-α, interleukin-6 (IL-6), and interleukin-1 beta, thereby attenuating ocular inflammation. Additionally, melatonin’s antioxidative properties play a crucial role in mitigating oxidative stress, a prominent feature in uveitis pathophysiology [166]. Interestingly, topical melatonin instillation also exerted a detectable effect. In a study using rabbits, acute immunogenic uveitis was induced by injections of normal horse serum. Researchers investigated the effects of applying a 0.1% melatonin solution on uveitis symptoms and biochemical markers in tear fluid and aqueous humor. They found that melatonin reduced uveitis symptoms and increased antioxidant activity in tears while lowering levels of α(2)-macroglobulin. A similar trend was observed in aqueous humor, with higher antioxidant activity and lower levels of protein and α(2)-macroglobulin in treated rabbits. These findings suggest that melatonin instillations enhance local antioxidant activity and reduce inflammation severity and permeability of the blood-ocular barrier in uveitis [167]. 

While the existing evidence is promising, human clinical trials are necessary to determine efficacy, optimal dosing regimens, treatment durations, and potential combination therapies involving melatonin for uveitis. Moreover, research focusing on the safety and efficacy of topical and intravitreal administration of melatonin is crucial for its translation into clinical practice.

In conclusion, the anti-inflammatory, immunomodulatory, and antioxidative properties of melatonin position it as a promising candidate for the adjunctive or alternative management of uveitis. The available literature supports the potential therapeutic role of melatonin in uveitis, underscoring the need for additional research to elucidate its clinical utility and optimize treatment strategies.

## 11. Melatonin and Retinitis Pigmentosa

The investigation into melatonin’s role in retinal health spans various studies, shedding light on its potential therapeutic benefits for retinal degenerative diseases like retinitis pigmentosa (RP). In a study on patients with night blindness, Banas et al. [168] found that damage to photoreceptors influenced melatonin secretion and its circadian rhythm. This observation underscores the link between retinal health and melatonin regulation. Preclinical studies using animal models further elucidate melatonin’s protective effects on retinal degeneration. Xu et al. [169] demonstrated that daily melatonin injections in rd10 mice, a model of autosomal recessive RP, significantly delayed photoreceptor loss and reduced inflammation-related gene expression. Similarly, Liang et al. [170] found that melatonin treatment in rds/rds mice slowed photoreceptor degeneration and reduced apoptotic cell death, suggesting its potential as a therapeutic intervention for RP. Recognizing the significant impact of RP on patients’ quality of life, Pastor-Idoate et al. [171] proposed a clinical trial to assess the efficacy of oral melatonin (OM) administration, alone or combined with short-wavelength light (SWL)-blocking filters, in alleviating sleep disorders and psychological stress in RP patients. This study aims to evaluate changes in hormone release, sleep quality, retinal function, and patient-reported variables, offering insights into the potential benefits of melatonin supplementation as a low-cost therapeutic approach for RP patients.

Overall, these studies highlight the multifaceted role of melatonin in retinal health and its potential as a therapeutic agent for mitigating retinal degeneration in conditions like RP. Further research and clinical trials are warranted to validate these findings and explore melatonin’s broader applications in preserving retinal function and improving patients’ quality of life.

## 12. Melatonin and Myopia

Myopia, a prevalent refractive error, poses significant risks of adverse outcomes. Most creatures, including humans, are initially inclined towards hyperopia. As the eye develops, it undergoes axial elongation, a process called emmetropization, influenced by visual stimuli. The surge in myopia rates and its correlation with education levels underscore substantial environmental influences on its development, despite the familial inheritance patterns that suggest a genetic component. Over 150 genetic syndromes include familial high myopia, indicating a genetic predisposition. Therefore, both genetic and environmental factors contribute to myopia’s etiology. Various pathways regulating growth, such as dopamine, ZENK-glucagon, retinoic acid, crystallin, serotonin, melatonin, vasoactive intestinal peptide, enkephalins, nitric oxide, and growth factors, are known to be involved in the control of eye growth and axial elongation [172].

The complex relationship between the sleep hormone melatonin and myopia has intrigued researchers for some time. While experimental models hint at a potential link between disrupted melatonin rhythms and myopia development [173,174], the true picture in humans remains shrouded in uncertainty. 

Several studies have addressed the assessment of melatonin levels and their daily fluctuations in diverse body fluids like saliva, blood serum, and urine among both myopic and non-myopic individuals. However, the findings presented a rather discordant symphony. While some studies reported higher, even significantly higher, systemic melatonin concentrations in myopes compared to individuals with normal vision (emmetropes) [175,176], others found no or even negative associations [177,178]. This inconsistency played out across different measuring methods and sampling times. Morning blood serum levels varied greatly between studies, with maximum and minimum values showcasing a wide range. Likewise, salivary melatonin patterns measured every 4 h over 24 h revealed no significant differences between groups, while hourly evening measurements hinted at potentially higher levels in myopes. Even the timing of melatonin release after dim-light exposure, known as dim-light melatonin onset, lacked a consistent connection to the refractive group across studies. A lone study offered a different perspective, reporting significantly lower levels of a melatonin metabolite in overnight urine samples from myopes compared to emmetropes [179].

In summary, while the exact role of melatonin in myopia remains unclear, the current evidence suggests a fascinating, yet complex, relationship. Further research with rigorous methodologies and controlled variables is crucial to harmonize the discordant notes and understand the true melody of melatonin’s influence on myopia.

Despite the promising findings reported here, the existing clinical trials and research studies are still insufficient, particularly for various ocular diseases and alternative drug administration methods. Further large-scale clinical trials are needed to determine the appropriate dosages, administration routes, and treatment time windows for melatonin in the context of ocular diseases [14]. However, we believe that—although the research data can be considered still in their infancy—they are paving a promising avenue for the development of a topical formulation of melatonin to treat or even prevent different ocular pathologies such as dry eye, glaucoma, cataract, and other retinopathies. The greatest challenge at this moment is to find a formulation that has three fundamental properties:

I. Stability (at least 3 years of shelf-life). In my personal experience (DR, unpublished), this result could be achieved with nanomicellar formulations either by storing the bottles at −20 °C, storing them under vacuum in a refrigerator, or by lyophilizing the complete formulation, to be recovered at the moment of use with sterile distilled water. Full biological activity will be re-established after recovering the formulation.
II. Permeability (ability to cross the ocular surface barrier and diffuse to inner eye tissues, up until the retina). Different nanotechnological formulations appear appropriate for this purpose.
III. Industrial feasibility and commercial value (the cost should be low enough to guarantee an affordable sale price). This kind of evaluation pertains, of course, to pharmaceutical companies’ management.

We hope that the information reported in this review could encourage research institutes and pharmaceutical companies to continue with this avenue and propose a product that could benefit millions of potential patients worldwide.

## 13. Conclusions

In summary, the scientific literature suggests that melatonin holds promise as a potential therapeutic agent for a range of ocular diseases, especially in the context of its antioxidant, anti-inflammatory, and neuroprotective properties. Therefore, it is a promising candidate for managing conditions such as age-related macular degeneration (AMD), glaucoma, diabetic retinopathy, and cataracts. Melatonin’s ability to scavenge free radicals and mitigate oxidative stress may help protect the retina and lens from damage. Additionally, its anti-inflammatory effects could be beneficial in reducing inflammation in different parts of the eye. Furthermore, melatonin’s role in regulating circadian rhythms may have implications for ocular health, as disruptions in circadian rhythms have been linked to certain eye disorders.

However, further extensive research and clinical trials are necessary to fully understand its efficacy, safety, and optimal administration methods for different ocular conditions. The topic administration of melatonin as eye drops remains a challenging, however, affordable objective, due to the instability of the molecule in aqueous solution, and the low efficiency of delivery to the posterior pole.

## Data Availability

Data sharing is not applicable.
